# Supplementary material for: Students’ negative emotions and their rational and irrational behaviors during COVID-19 outbreak
Source: PLoS One. 2022 Mar 7;17(3):e0264985. doi: 10.1371/journal.pone.0264985 (PMC8901067; doi:10.1371/journal.pone.0264985)
Supplement: Attachment — Submitted filename: Reviewer-Final (1).docx [file pone.0264985.s001.docx]

December 28, 2021

To: Editor-in-Chief,

PLOS ONE, [PONE-D-21-32396]

Dear Editor,

We would like to thank the reviewers for useful suggestions and constructive comments on our manuscript PONE-D-21-32396. We believe that the comments helped us to enhance the quality of the manuscript. We have read the referee remarks attentively and try to amend the manuscript to address all the comments properly. Please find enclosed our report to the comments of the referees and the revised version of our manuscript " Emotions and students rational and irrational behaviors, a COVID-19 case study" by M. Rezapour et al.

We have taken into account of all the comments and suggestions of the reviewer(s) point by point to improve our manuscript. We believe that we have adequately addressed all the possible questions and concerns. We look forward to your review and your prompt reply

Best regards,

M. Rezapour, A. Dehzangi, F Saadati

**Reviewer #1:**

In the current research article entitled "Emotions and students rational and irrational behaviors, a Covid-19 case study", by Rezapour, conducted by using the results of a survey conducted across 2,534 students enrolled in 7 states in the US. Various feelings such as sad, irritable, stress and guilt were analyzed in a unified model by means of seemingly unrelated regression. it was found that having a higher body mass index (BMI), self-rated worse health conditions, and limiting outdoor activities would have detrimental effects on the mental health of students. This article addresses a research topic of great interest, which is under intense investigation in the past years and the manuscript is generally well-written. However, this reviewer has certain suggestions that would help produce a more comprehensive overview of the topic:

Suggestions: -

1) The authors should cross-check all abbreviations in the manuscript. Initially, define in full name followed by abbreviation.
Thanks for catching the errors on abbreviations. We went through the whole manuscript and made extensive amendments. Special attention was made to make sure the abbreviations were spelled out before their first use.

2) The English of manuscript can be polished (minor).

We agree and we had the manuscript polished to reflect better English writing. Some of the corrections can be seen from the highlights. Some minor corrections were made without the highlight showing.

3) The authors may additionally provide one Figure as summary, challenges, or prospect of the present study.

That is an excellent suggestion and accordingly, Figure 1, and the necessary description was added to the manuscript to address the comment. Please refer to Figure 1,a,b,c in the manuscript.

**Reviewer #2:**

In this paper entitled " Emotions and students rational and irrational behaviors, a Covid-19 case study", the author's goal is to conduct an in-depth evaluation of associations between various emotions that students experience during COVID-19 pandemic". The manuscript is easy to understand and data-oriented. However, it requires few corrections.


Minor Comments:

1) The English may be polished. There are grammatical errors and spelling mistakes in the manuscript.

We agree and we have the manuscript polished to reflect better English writing. Some of the corrections can be seen from the highlights. Some minor corrections were made without the highlight showing.

2) Discuss studies related to the manuscript in the introduction.

Thanks for the reminder, to address the reviewer comments we added related studies to the manuscript. The introduction was extensively amended and also a section of “Literature Review” was created and several references were added and relevant studies were covered. Please visit the “Introduction” and “Literature Review” sections all the changes are emphasized in revised manuscript with highlights.

3) What are the criteria for selecting the emotions incorporated in the study.

That is a nice point and added in the data section as”

*….“Finally, it should be noted that the criteria for selection of various responses for different models are due to specific question related to different negative emotions. In other words, all negative emotions highlighted in the survey were incorporated as our response for the statistical modeling.“…*

Regarding our selection of quantitative impacts to measure, we chose nine survey items based on information gathered from all the dat. These nine survey items measured the following concepts: negative emotion states, preoccupation with COVID-19, feeling stressed, worry, and time demands. Negative emotion states comprised four of the survey items. Each item explained one of the basic negative emotions (i.e., being afraid, irritable, guilty, and sad) identified during the development of the PANAS.

We also amended the text as following for more clarity. Please visit the “Methodology and Data Analysis” section:

*… ”* *For the quantitative assessment, nine survey items were chosen based on information gathered from all the data, and all the concepts of interest were measured from these survey items. The negative emotion states were four items of those, where each item was dedicated to each of the negative emotions. These negative emotions for each survey item were recognized during the development of the positive and negative affect schedule (PANAS) [29]. We had different emotions that the respondents experienced due to COVID where all of those emotions were considered. In the data analysis, these negative emotions including being sad, afraid, irritable, stressed and guilty, with similar scale, were used..”….*

4) Discussion: The information about mortality rate and various prevention approaches should be provided related to immunity, and health, and influence of COVID-19 variants i.e. doi: 10.1007/s12088-020-00908-0; doi: 10.1007/s12088-020-00893-4, and doi: 10.1007/s15010-021-01734-2.

The suggested resources were considered and partially added to the “Introduction”.

*….“So far extensive works have been employed for various aspects of COVID-19, from immunity [5], to its variant [6].”……*

5) Mentions the tools used during the dataset analysis in the Data section. Also, highlight how the present manuscript is different from studies done using the same data set.

We added the following paragraph to address your concern:

*……”It should be noted compared with the previous study which used the similar dataset [27], we employed a unified technique to account for all types of negative emotions, instead of focusing on a single one. Also, the previous study focused on the structure of the variables by means of factor analysis, while we focused mainly on the impact of various variables in a unified model. The vector generalized linear model (VGLM) function in R was used for conducting the statistical analysis [30].”……..*

6) The author should provide at least one or two illustrations (additional Figures) to highlight the summary and significance.

That is an excellent and relevant point, which was also suggested by the other reviewer. We included Figure 1 to address the comment.

7) Advanced statistical tools can be used to analyze the study comprehensively.

We agree that there are many possibilities techniques and statistical tools that could be used to model the impact of the pandemic. We tried to choose the best applicable tool relevant to our objectives to model equations related to different negative emotions simultaneously. We definitely will try to use more advanced tool for our future study, but at this time given to our resources and limitations, the implemented technique seems our only viable one.

**Reviewer #3**: 
The authors report a case study titled “Emotions and students rational and irrational behaviors, a Covid-19 case study”. This study uses a survey-based approach along with statistical analyses to analyze the effects of Covid-19 pandemic on the mental health of students. A total of 2534 students participated in this study. Feelings such as sadness, irritability, stress and guilt were analyzed in this study. The topic of study is very relevant and interesting to the general audience.


Major Comment:
According to the National Center for Education Statistics (NCES), 19.7 million students were enrolled at U.S. colleges in fall 2020. Among them there were approximately 16.7 million undergraduate students and 3.1 million graduate students. States like California, Texas, Illinois, Minnesota enrolls a significant population of students. Additionally, these states represent the diverse demographics (location, weather, climate etc.) of the USA, which could influence the emotions among students. Unfortunately, the authors have not provided any data from these states. Assessing the emotions and behavior could be complex and hence several factors need to be considered. A study by Kim et al. (10.3390/ijerph14040431) suggested that both demographic and environmental factors can influence mental health which translates to behavior. In light of the above reasons, I believe the current study is limited by the number of students participated as well as location. The authors could include more participants from schools of Mid-western states and bay areas (like California).

Thank you very much for pointing out the demographics. We agree that the diverse demographics could add deeper prospect to the present study. More study yet to be conducted on the case to prevail a comprehensive overview of the impact of COVID-19 on our students. The main focus of this research was just to put some light on the subject of negative emotions and their rational and irrational behaviors during COVID-19 for the college students of specific universities, and we don’t claim delivering a comprehensive study. To address the limitation of our study, multiple resources were cited and we incorporated them as the limitation of our study in different part of manuscript. More details about the study were added to manuscript for instance look at the following sentence at the first paragraph of “Methodology and Data Analysis”:

*…” Out of those, 2,534 responses were obtained and used [27]. From those responses 61% were female compared with 39% being male, also 77% were younger students (i.e., 18-24 years old), and the rest were older. “….*

At this time, it is not practical for us to recollect the data as the circumstances have varied. However, to address the reviewer concern, we included the following statement to discuss the limitation of this study to the “Discussion” section:

….” *Due to the universities closure, we used a convenience sampling (or nonprobability sampling) method, which may affect the results. It means the profile of this sample may be a biased representation of the US college students in general. Therefore, a precaution should be given while interpreting and using the findings. In addition, only specific locations with unique demographic characteristics, e.g.., location, weather, climate, were incorporated. Emotions and behaviors are expected to be complex [54], thus other possible factors related to the above points should be considered. As the results of the survey were collected in spring 2020 when the first and most severe real lockdown occurred in the U.S., it is not practical to recollect the data to incorporate more observations.*

*It is worth noting that, having students from other parts of the country - such as southern states of the US - could have brought more reliable outcome for present study. Therefore, in order to make sure about the generalizability of these results, future research could focus on double checking the results with larger and randomized sample of students from more states and universities.*

*For future direction of research, more investigations such as in-person interviews can be considered to highlight the justification behind taking unnecessary precautionary behaviors, e.g., limited exercise at home. That way, our hypothesis regarding impaired emotions of students could have been confirmed. Despite the limitations, we were able to highlight the associations between various variables and negative emotions, especially during life-changing pandemic of COVID-19.*”….
